# Supplementary material for: The microbiome biomarkers of pregnant women’s vaginal area predict preterm prelabor rupture in Western China
Source: Front Cell Infect Microbiol. 2024 Oct 31;14:1471027. doi: 10.3389/fcimb.2024.1471027 (PMC11560878; doi:10.3389/fcimb.2024.1471027)
Supplement: Supplementary file 1 [file DataSheet1.zip › compare_1/Community/Pieplot/family/group/C.family.pieplot.pdf]

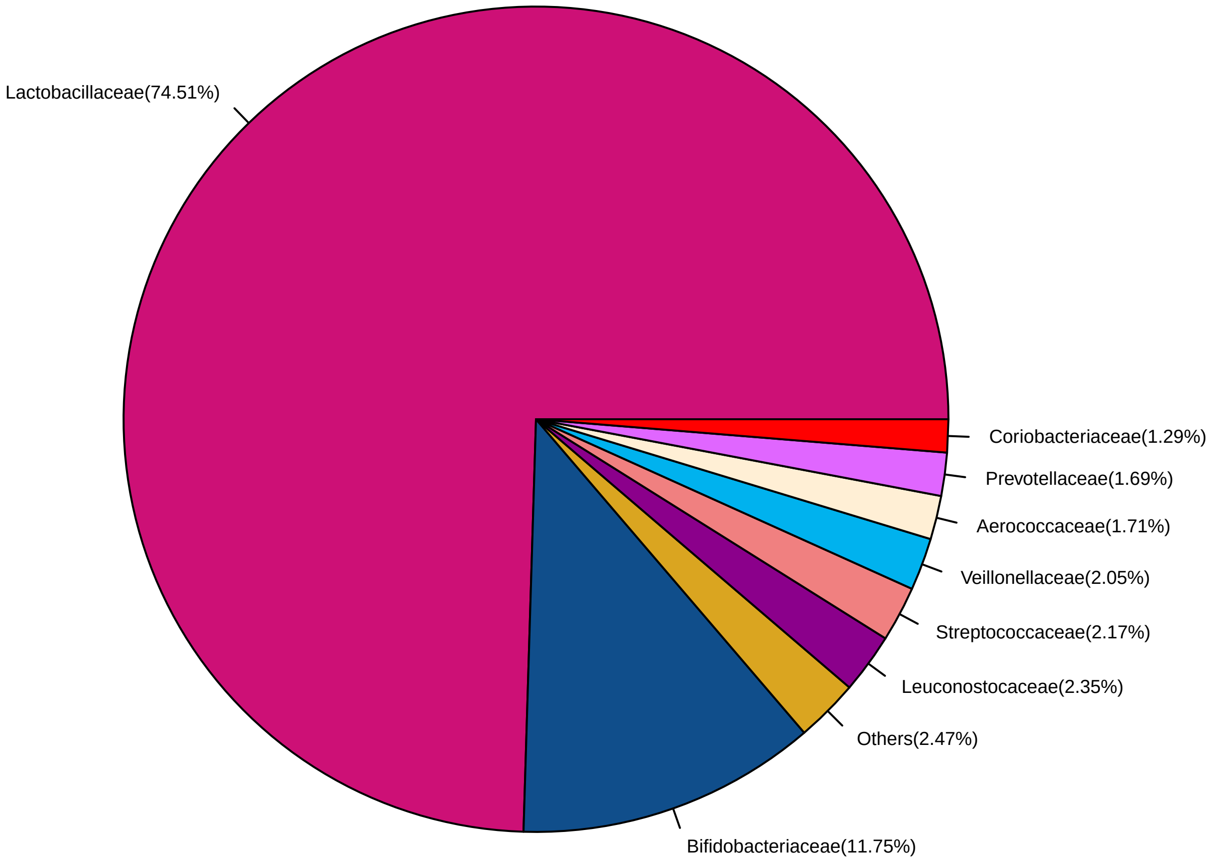
Lactobacillaceae(74.51%)
Coriobacteriaceae(1.29%)
Prevotellaceae(1.69%)
Aerococcaceae(1.71%)
Veillonellaceae(2.05%)
Streptococcaceae(2.17%)
Leuconostocaceae(2.35%)
Others(2.47%)
Bifidobacteriaceae(11.75%)
